# Using the Trauma Reintegration Process to Treat Posttraumatic Stress Disorder with Dissociation and Somatic Features: A Case Series

**DOI:** 10.3390/healthcare13101092

**Published:** 2025-05-08

**Authors:** Mary T. Sise

**Affiliations:** Independent Researcher, Latham, NY 12110, USA; msise3@gmail.com

**Keywords:** emotional freedom techniques, EFT, tapping, trauma reintegration process, TRP, dissociation, post-traumatic stress disorder, PTSD, case series

## Abstract

Given the suboptimal responses to medication and cognitive behavioral therapies in the treatment of post-traumatic stress disorder (PTSD), new approaches are needed. **Background/Objectives**: Therapies that include a somatic component such as Emotional Freedom Techniques (EFT) and Eye Movement Desensitization and Reprocessing (EMDR) have demonstrated efficacy in the treatment of PTSD in numerous clinical trials. This case series introduces the Trauma Reintegration Process (TRP), a psychotherapeutic process developed by the author that can be combined with somatic therapies to enhance their effectiveness, especially in patients with dissociation. **Methods**: This case series describes the use of TRP in combination with EFT, an energy-based somatic treatment that engages the meridian system of the body through gentle tapping on acupressure points. TRP uses EFT in combination with a focused guided imagery sequence. This case series describes the treatment of two patients: a 20-year-old woman who experienced PTSD and somatic symptoms following a serious motor vehicle accident (MVA) and a 45-year-old woman with a history of severe abuse as a child as well as adult trauma who had also been in a serious MVA. The cases contrast the way TRP can be applied in patients with single versus multiple traumas and who experience dissociation. **Results**: In both cases, EFT treatment stalled when the patient dissociated. After TRP was introduced, however, the EFT treatment regained momentum, leading to significant improvement in PTSD symptoms including a reduction of nightmares and flashbacks and resolution of other somatic symptoms. **Conclusions**: The trauma reintegration process (TRP) in combination with EFT has the potential to assist in the memory processing of patients with dissociation and complicated trauma presentation without retraumatizing the client and causing further distress or dissociation. In addition, it provides the patient with a self-empowering method to alleviate any additional traumatic sequelae.

## 1. Introduction

Posttraumatic stress disorder (PTSD) is a psychiatric disorder that may occur in people who experience or witness traumatic events or circumstances that are physically or emotionally harmful or life-threatening, including but not limited to natural disasters, serious accidents, war/combat, sexual assault, intimate partner violence, and bullying [1]. Up to 70% of adults have experienced at least one traumatic life event, and it is estimated that 20% of those who experience trauma will develop PTSD [1]. The American Psychiatric Association [2] defines PTSD in terms of four groups of symptoms: (1) reexperiencing symptoms, such as flashbacks, nightmares, and frightening thoughts; (2) avoidance symptoms; (3) hyperarousal symptoms, such as startle or jitteriness; and (4) negative cognition and mood symptoms, which are negative changes in beliefs and feelings.

PTSD exerts an enormous economic burden, driven by disability, health care, and unemployment [3]. It affects approximately 3.5% of U.S. adults every year [4]. It is estimated that over 7 million people in the United States suffer from PTSD and less than half of those experiencing PTSD receive professional help [1]. Even among individuals actively receiving psychiatric care, treating PTSD remains challenging, with up to half of patients continuing to experience persistent symptoms despite therapy and medication [5]. For instance, though recommended by PTSD treatment guidelines [6], cognitive behavior therapy (CBT) has limited effectiveness. In a comprehensive study published in the *Journal of the American Medical Association*, approximately two thirds of 891 service members and veterans completing a course of CBT as part of peer-reviewed studies published between 1980 and 2015 still met PTSD diagnostic criteria after treatment [7]. A more recent systematic review (2020) reports average CBT dropout rates of 15–20% and highlights variability in treatment response [8]. With the demand for psychiatric care to treat PTSD exceeding the supply, the development of easily applied treatments that can also be used on a self-help basis is critical.

The importance of bringing a somatic component into the treatment of PTSD has received growing recognition since van der Kolk’s classic paper “The Body Keeps the Score” [9] and subsequent book of the same title [10]. Among the psychotherapies that have been shown to be effective for PTSD are somatic experiencing [11], Eye Movement Desensitization and Reprocessing (EMDR) [12,13], and Emotional Freedom Techniques (EFT) [14,15,16,17]. Since EFT was the treatment received by both cases in this case review, EFT will be our focus.

## 2. Methods

### 2.1. Emotional Freedom Techniques

Emotional Freedom Techniques (EFT), commonly called “tapping”, is a widely practiced and systematically researched treatment approach within the emerging area known as energy psychology [18]. Evidence of EFT’s effectiveness for a range of physical and psychological conditions has been documented in more than 120 clinical trials [19]. Developed in the late 1990s by Gary Craig, EFT is a derivative of thought field therapy [20,21]. It involves focusing on a disturbing thought, traumatic event, or belief while activating the energy meridian system of traditional Chinese medicine by gently tapping with the fingertips on specific acupuncture points (acupoints). Currently, there are over 40 different versions of tapping and several training organizations [19]. While a single clinical protocol (Clinical EFT) is in wide use in research settings, the approach can be integrated into a range of clinical protocols [17,22].

**Figure 1:**
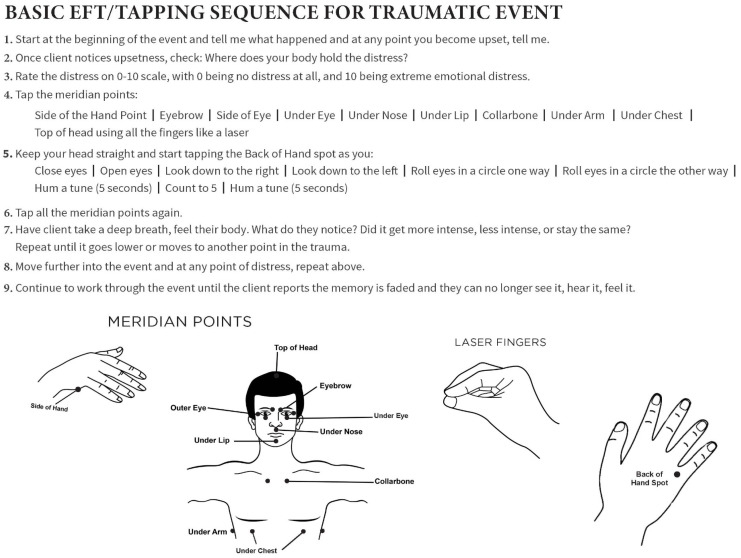
Basic EFT/tapping sequence for a traumatic event.

Figure 1 details an EFT tapping protocol for trauma. Due to its simplicity and the quick relief it offers, EFT is increasingly popular as both a powerful modality for self-help and as a tool for processing trauma under the direction of a licensed mental health practitioner. Informed estimates suggest that tens of thousands of clinicians are using the method with their clients [23] (p. 18) and “tens of millions” of individuals are using EFT worldwide [24].

EFT builds upon established clinical methods (psychological exposure and cognitive restructuring) while adding the stimulation of selected acupuncture points by tapping on them. Mounting evidence suggests that acupoint tapping sends clinically beneficial activating and deactivating signals to brain areas that were aroused during the cognitive and exposure aspects of the protocol [14,15]. The core hypothesis to explain EFT’s consistently strong clinical outcomes is that tapping on acupoints generates electromagnetic signals that have a therapeutic effect on areas of the brain involved with distress, trauma, or mental illness [15].

In a comparison study by Mavranezouli et al. [25], EFT was one of the two most effective therapies in reducing PTSD symptoms at treatment endpoint and the most effective of the 17 interventions in retaining improvement in PTSD symptoms on follow-up.

In 10 studies comparing tapping to CBT for a variety of conditions, all 10 found at least equivalent outcomes, and in several studies, the tapping protocols outperformed CBT in speed and durability on follow-up [26]. Research has shown EFT to be effective for PTSD symptom remediation in veterans [27,28,29] and results can be achieved rapidly. In the Church and Brooks study, the beneficial effects were seen in only six sessions, and the Geronilla et al. study results were seen in 5 to 10 sessions. This is contrasted with a range of 12 to 15 sessions for trauma-focused CBT in typical cases, extending to 16–25 sessions for complex trauma [30].

Figure 1 shows a basic tapping sequence for traumatic events. Note that in this author’s application of emotional freedom techniques (EFT), traditional setup statements and verbal reminder phrases are intentionally omitted during the treatment of traumatic material. This modification arises from clinical observations that verbal components, while useful in some contexts, may inadvertently heighten cognitive activation or re-evoke traumatic imagery. Instead, the process emphasizes continuous somatic engagement through tapping on the meridian endpoints. By anchoring attention in the body rather than in narrative recall, this approach supports physiological regulation and reduces the risk of dysregulation, flashbacks, or retraumatization. The rhythmic stimulation of acupoints serves not only to discharge the distressing affect, but also to maintain a present-moment focus, which is particularly vital for clients with a history of dissociation or panic responses.

### 2.2. Trauma Reintegration Process

The Trauma Reintegration Process (TRP) is a method developed by the author to address the dissociative symptoms and fragmented consciousness often seen in traumatized clients. The *Diagnostic and Statistical Manual of Mental Disorders* (DSM-5) defines dissociation as a disruption, interruption, and/or discontinuity of the normal, subjective integration of behavior, memory, identity, consciousness, emotion, perception, body representation, and motor control [31]. Examples of dissociative symptoms include the experience of detachment or feeling as if one is outside one’s body and loss of memory or amnesia [32]. Dissociative disorders are frequently associated with previous experience of trauma [33]. According to van der Kolk [10] (p.66): “Dissociation is the essence of trauma. The overwhelming experience is split off and fragmented, so that the emotions, sounds, images, thoughts and physical sensations related to the trauma take on a life of their own”.

TRP was developed to assist clients whose dissociation interfered with the progress of the primary treatment modality, including EFT and EMDR. TRP draws on over 30 years of clinical experience with trauma survivors with severe dissociative disorders. When used in conjunction with EFT or other somatic interventions, it allows the client to experience the emotions the dissociated part is holding, use the other therapy intervention to lower their distress, and then reunite them with the other parts, thus moving the trauma story to completion.

TRP integrates guided imagery with principles drawn from traditional healing practices, including elements akin to soul retrieval. One difference between TRP and other energy-based methods, such as Matrix Reimprinting, is that TRP does not attempt to rescript the historical event or alter others’ behaviors within the memory. Crucially, the retrieved part is not named or assigned a fixed identity; it is regarded as an aspect of consciousness, intentionally avoiding the crystallization of that energy into a separate persona. While the part may be perceived as childlike, TRP refrains from reinforcing developmental roles or imposing narrative labels.

Another distinction is that TRP culminates with the client “looking out the eyes of today”, symbolizing the energetic integration of past and present selves. Clinically, clients report a profound shift following this integrative act, often describing an embodied sense of fullness or wholeness, accompanied by the resolution of longstanding feelings of inner emptiness or fragmentation.

TRP has been taught to hundreds of therapists through workshops and training sessions. The steps of TRP are outlined in Table 1 and explained in each of the cases. Three Supplemental Tables provide additional examples of the Trauma Reintegration Process, outlining how it can be used in cases of sexual assault, childhood abuse or neglect, and with combat veterans.

### 2.3. Adverse Childhood Experiences Questionnaire

The adverse childhood experiences (ACE) score derives from a self-report questionnaire used widely to assess the impact of adverse childhood experiences on health and well-being in adulthood [34]. The questionnaire comprises 10 items assessing exposure to different forms of abuse, neglect, and household dysfunction before age 18. Responses are given on a binary scale (yes or no) to each item (e.g., “Did a household member go to prison?”). The score range for the ACE questionnaire is 0 to 10, with higher scores indicating greater exposure to adverse experiences. Survey data project that approximately 61% of adults in the U.S. have experienced at least one category of ACE and 17% have experienced four or more [35]. A review of the impact of energy psychology protocols such as EFT on the regions of the brain most centrally involved with multiple or severe adverse childhood experiences came to the following conclusion: “Energy psychology protocols, with their use of acupoint tapping, show promise for enhancing the effectiveness of more conventional clinical techniques” [36].

### 2.4. Combining the Methods

The following cases illustrate how TRP can be integrated with EFT in the treatment of PTSD. Both patients presented with persistent somatic symptoms triggered by a motor vehicle accident (MVA). In each case, the patient experienced dissociation during the trauma and was therefore unable to access the distress when recalling it in the treatment session. These cases also contrast treatment delivered to patients with and without preexisting childhood trauma. ACE score questionnaires were used in both cases to inform the clinician of preexisting trauma, as this impacts the treatment plan.

These cases were chosen in particular to show the applicability of TRP with both single and multiple incident traumas. The methodology of EFT was used because the clients were comfortable with this method and could also apply it at home in a self-empowered way should further aspects of the trauma surface. In addition, for clients with intense panic, being able to titrate the emotional response with EFT is critical to helping them not to relapse into a dissociative or freeze response, have a flashback, or feel retraumatized.

## 3. Results

As an exploratory case series, this research does not include standardized pre-/post-assessment measures.

**Case 1: MVA in a young woman without a trauma history**

L is a 20-year-old college student who sought therapy to help manage chronic pain symptoms. L reported one adverse childhood experience, which was that her mother was sick in bed, depressed, and unavailable for periods of time to take care of L and her sister (ACE score = 1). During the time of treatment, she received a diagnosis of Ehlers-Danlos syndrome and, with EFT and physical therapy, experienced improvement in her functional status.

During the COVID-19 pandemic, L returned to care, attending a Zoom online group focused on EFT. Lying in her bed, she told the group of 80 participants that she had been in a serious MVA 10 weeks prior and had since been confined to her bed due to debilitating pain and inability to walk. She reported that immediately after the accident, she was able to walk; however, 24 hours later she was “frozen in her bed” and her parents had to assist her with all activities of daily living in the confines of her bed, including carrying her down the stairs to attend doctor’s appointments. She reported nightmares, intrusive thoughts, and an inability to move. She had received medical care after the MVA, and extensive diagnostic evaluation found no injuries.

As we began to process the trauma of the MVA, L revealed the moment-to-moment details of the accident. At each point where she experienced distress, she rated it using the subjective units of distress (SUD) scale, with 0 being no distress and 10 being the greatest distress [37]. We then used EFT to reduce the distress. Initially, each aspect of the memory became less intense as she completed each round of EFT (a round consists of items 4–6 in Figure 1).

L then began to describe the experience of being trapped in the car for one hour until the emergency workers could free her from the vehicle. During the recounting of this part of the memory, the client described having no feelings and EFT did not impact the SUD level. When she looked at that part of the accident where she was trapped in the car, she felt nothing. She was numb. Recognizing this, the therapist had her begin the TRP (outlined in Table 1) to access the part of her that had split off (dissociated).

1. I asked, “Where do you see yourself?” L replied she was “outside the car watching herself stuck inside the car”.
2. I guided her to imagine the her of today going to the part of her that was outside the car and first comforting her. As she imagined herself approaching the part of her outside the car, she began to feel some anxiety. She imagined herself putting her arms around this aspect of her. As she did this, she began to feel warmth in her body. We did a round of tapping, the SUD level lowered, and she no longer felt disconnected from that part. I then instructed her to move closer to the part of her still in the car and notice what sensations or emotions were happening in her body.
3. As she imagined herself returning to the car, she reported that she could now feel herself inside the car feeling frozen and trapped, stating, “My heart is racing and I feel panicked”. We then quickly used EFT to reduce the panic. Once all emotional aspects of the memory had been addressed and the SUD score lowered, I then instructed her to tell herself the truth, that she was freed from the car, that she and her mother had survived the accident, and she was now safe. She did this and repeated EFT. Her body relaxed and her SUD score decreased. The memory became less vivid, the pounding in her chest stopped, and she could experience the next part of the event of being removed from the car by the workers.
4. I invited her to bring herself from the MVA forward to the present and to look out her eyes and see today. We used a final round of EFT. She reported a sense of deep physical relief in her body.

To assess her progress, I asked L to go back and remember the MVA. She reported she no longer felt stuck in the car. As the images faded, the emotions and panic released. She finally felt a sense that the MVA was over, and she was safe again. At this point, I invited her to see if she could move. She surprised me and the entire group on the call when she was able to get out of bed and walk across the room unassisted. This dramatic improvement in her somatic symptoms was later confirmed by her parents. Two years later, L reported sustained, significant improvement, returning to college shortly after our session and resuming her normal physical and extracurricular activities, with limited pain.

L offered the following assessment of her treatment: “Being in a car accident is a scary moment on its own. Not being able to move, having to rely on people around you for anything you need to do, having no medical explanation for your symptoms, and being told by doctors, ‘I see nothing wrong,’ it’s even scarier, because you don’t know how to heal, if you’ll heal, or if you’ll ever be a normal teenager again. At first, I didn’t believe tapping could really help me, and when Mary told me to try to get up and walk, I was scared to get hurt, but it worked and it felt like a true miracle. Within the next few weeks, I was able to get my driver’s license and travel halfway across the world to attend college classes. During the last three years, pain began to decrease slowly. I took things slowly, not putting too much pressure on my body, but now I feel in amazing shape. I still use tapping in periods of stress or when I feel down emotionally. I tap on my hands, on my body and on my face, if I’m not in a crowded place, and it helps me get back on my feet stress-free”.

**Case 2: MVA in a woman with an extensive trauma history**

M was 45 with a history of childhood and adult trauma that included neglect, physical abuse, sexual assault, and domestic violence. She was referred for evaluation of worsening PTSD symptoms after an MVA. Her prior therapist referred her for a second opinion and consideration of EFT after M experienced a sexual-assault flashback during a therapy session. Neglected by her mother, who suffered from depression, and physically abused by her father, M, at age 19, was repeatedly sexually molested by a male college professor, which she did not disclose at the time; M’s history included a one-month psychiatric hospitalization for depression and catatonia (ACE score = 6).

M had left a physically abusive marriage six years prior to our session, and she had an elementary-school-aged son. Despite her trauma history, she had been stable in monthly therapy for seven years prior to the MVA and was happily employed in special education. She had received a diagnosis of major depression and panic disorder, and throughout her therapy, she tried numerous psychotropic medications with little relief. After the MVA, she began experiencing panic attacks, nightmares, and flashbacks to her earlier childhood traumas and sexual assault. She also began experiencing a constellation of physical symptoms including muscle pain, insomnia, and brain fog. These worsened with time and, two months after the MVA, she was diagnosed with chronic fatigue syndrome and was experiencing chronic musculoskeletal pain. She was using a walker for short distances and a wheelchair for longer distances and reported loss of visual acuity. These disabilities led her to be unable to work. At the time of referral for EFT, she was experiencing dissociative symptoms and reported several instances of not knowing where she was. She reported feeling desperate, with passive thoughts of suicide; however, she did not have a plan and lacked active suicidality due to the need to care for her son.

Because of the significant amount of childhood neglect and trauma in M’s history, her treatment was first focused on stabilization strategies and coping skills prior to trauma processing. First, we used EFT to calm her panic symptoms. This served to lower her SUD level such that she felt some control over her bodily reactions and day-to-day functioning. She was instructed to use EFT throughout the day, on a daily basis, whenever she felt frozen or terrified. While she was unable to reach 0 SUD level at this point, she could see the progress of taking panic from a 10 to a 6 level and this empowered her to continue the work. Second, we used EFT to create safety for her. We added visualizations for her to use in everyday life [38] (pp. 237–257), such as applying for disability, speaking up at a medical appointment, and lowering her distress about completing activities needed to care for her son. She would first envision what she desired and then use EFT to reduce any fear around that task. With this, her dissociative symptoms lessened considerably. Her startle reflex lessened, she used EFT after waking from nightmares, and these decreased in frequency. Slowly, she felt her physical health improve and she returned to walking independently.

After stabilization, we began to process the childhood traumas that were still being triggered. In her case, needing to ask for help after her MVA led to intense panic. We used EFT to process this and other triggers. Her SUD score decreased only by a few points. I then instructed her to notice the terror in her body and to go back to a time when she felt the same terror. We used TRP to improve the effectiveness of EFT while processing traumas that included dissociation (see Table 1 and Supplemental Table S1). She remembered one of her most traumatic memories when she had been abandoned by her mother at age 7 and left home alone with two younger siblings. Next, I asked, “Where do you see yourself?” (Step 1). She saw herself looking out the window, seeing her mother walk away into the woods. I invited her to imagine going closer to her younger self, and we used EFT to reduce the panic (Step 2). It took several rounds of EFT until she could actually get close enough to have a conversation with her younger self without feeling terror. At this point, I instructed her to comfort her younger self and tell her the rest of the story, that her mother returned and that she and her siblings were safe. She was able to comfort the child part of her and express anger at her mother for abandoning her. When the processing of this traumatic event felt complete, I instructed her to walk out the front door of the home and envision this part of her coming into the present day (Step 3). During this, we showed the younger self that she was safe and focusing on joys (parenthood). Once she was up to the present time, I instructed her, “Open your eyes and invite your younger self to look out your eyes and see today”. We repeated another round of EFT until she felt calm in her body and her SUD score was 0.

When contacted about this paper, M was honored to have her story shared and offered the following assessment of her treatment: “My life today seems like a miracle to me. When I first started learning tapping, I felt desperate because I was so physically ill and my body was breaking down from the stress of a chronic illness. I had no idea of the impact of my past on my health and ability to function. I just thought this was going to be my life: I was dying, and there was no hope for recovery. Once I learned tapping, it gave me hope that I could get my health and my life back. It was a long slow process with setbacks at times, but I could see I was able to do something specific to calm my nervous system and feel better. It was literally in my hands. When we were able to access my inner child and bring her to today, I felt such a sense of connection, wholeness, and joy. Today, at age 75, I am able to work part time to supplement my Social Security income. I no longer dissociate, rarely have nightmares, and can recover from upsetting events with much more ease. I volunteer regularly, enjoy babysitting my grandkids and singing in music groups. I attend a weekly online group tapping program when my schedule allows, and no longer need individual therapy. I no longer take any psych medicine. I am so very grateful and wish the whole world knew about tapping and bringing their parts back home”.

## 4. Discussion

In the two cases reported here, both patients experienced PTSD after a MVA and had prominent somatic symptoms, and each experienced dissociative symptoms. In the first case, the patient lacked a history of significant childhood trauma. When this is the case, the clinician can quickly begin to work directly on the trauma using EFT. The second case highlights the impact of preexisting trauma on the approach to treating PTSD symptoms. In this case, the whiplash injury from the MVA triggered reexperience of numerous prior traumas, including physical abuse and neglect as a child, domestic violence, and rape. Patients with extensive trauma history require a period of stabilization and safety prior to trauma processing. This is consistent with the practice guidelines of the International Society for Traumatic Stress Studies, which emphasize the importance of interventions to ensure safety and enhance personal, social, and environmental resources and self-regulation capacities prior to interventions for trauma memory processing [39] (p. 374).

The first step with M (Case 2) was to teach her how to use EFT to manage symptoms of panic and terror occurring in daily life. This empowered her and gave her hope. During this period, her SUD level would usually go from 10 to 6 but not any lower. To help demystify the PTSD symptoms she was experiencing, she was given information about the effects of trauma on the brain. Sessions were focused on daily functioning, taking care of her son, and improving her physical wellbeing. EFT was used consistently to allow her to visualize what she wished to do and to reduce anxiety. This enabled her to take new action. It required three years of twice-monthly sessions, combining stabilization and empowerment prior to working directly on her traumatic events. It enabled her to feel safe in the relationship with the therapist.

In both cases, the effectiveness of EFT stalled (the SUD level would not lower) when the patient experienced dissociative symptoms. When the effectiveness of EFT stalls during processing of the trauma, the clinician should ask questions that probe for dissociation, such as “Where do you see yourself in the story?” and “Are you looking on from above the accident/attack?” Probing the patient’s point of view can help determine the occurrence of dissociation, as the client typically reports that they see themselves separate from themselves and cannot feel, see, or smell what is happening. The next step in the work is to use guided imagery to guide the patient back into their body within the context of imagining the trauma and to feel the body sensations for a few seconds while using EFT to lower the terror and process the traumatic event. This frees the body to release its stored trauma. As van der Kolk [10] (p. 21) states: “This reintegration is necessary, as the body needs to learn that the danger has passed and to live in the reality of the present”. Clients consistently report that the Trauma Reintegration Process enables them to return to the present time and that when they revisit the traumatic story, they no longer feel stuck there. Clients typically report that the most powerful part is in the final step of the process, when they are guided to bring the dissociated parts to the present day, look through their own eyes, add a completion/safety affirmation (e.g., “I am safe”), and then do a round of EFT.

Another benefit of TRP, especially for clinicians working in outpatient settings, is that for clients who have multiple traumatic events with intense body memory such as gang rapes or sex trafficking, it is easier for the clinician and client to work with one incident at a time and not be flooded using EFT. With EFT, the client also has a tool they can use at home if other traumas intrude between the clinical sessions.

This report has the limitations of any case reports. The degree to which the outcomes can be generalized is unknown. In addition, no standardized pre-testing and post-testing of PTSD was utilized, only clinician observations and client reports.

## 5. Conclusions

Research supports the effectiveness of somatic therapies such as EFT for PTSD. The addition of the Trauma Reintegration Process (TRP) to established PTSD treatments offers an effective strategy when patients dissociate. Future research on the combination of TRP with other PTSD treatments will further explore the effectiveness of the method and how it may be most effectively applied.

## Figures and Tables

**Table 1:** Adding the trauma reintegration process (TRP) to EFT for dissociation.

	Steps for EFT Practitioner	Examples of Client Responses
**Step 1: Remember**	Ask questions to probe the client’s point of view.	Therapist: Where do you see yourself? What are you noticing? Client: I am outside the car, I am above the car looking down on myself stuck inside the wrecked car.
**Step 2: Reunite**	Guide the client to imagine getting as close as possible to the part of themselves they see still stuck in the trauma. If the SUD score increases, use EFT. Ask the client what they feel, hear, and smell. Guide the client through as many rounds of EFT as needed to reduce SUD score to 3 or less, moving closer and closer to the younger part, explaining and comforting the dissociated self with the truth of how the trauma is now over.	Therapist: Bring the you of today who knows you have been rescued from this accident first to the part outside the car. Use EFT. Comfort her and bring her to the part in the car. What do you see, feel, smell? Client: I can feel the terror now. Therapist guides client to do another round of EFT. Client: I feel calmer now, I see the rescue workers releasing me. I am now outside the car, I am shaking with fear. Therapist guides client through another round of EFT. Therapist: Comfort her and explain how you survived the accident. Client: I survived, I am ok, Mom also survived, I am safe now. Use EFT until SUD score is 3 or less.
**Step 3: Reintegrate**	After the SUD level has lowered to 3, invite the dissociated part (the younger self) to move forward to today and to look out of the client’s eyes in the present day. Repeat EFT with a calming statement until the SUD score is 0.	Therapist: With eyes closed, bring the you from the accident forward to today. When she is ready, invite her to look out your eyes, look around the room and see today. Client: I am ok now, my body is safe. I survived. Use EFT until the SUD score is 0 and the client is fully present.

## Data Availability

No new data were created or analyzed in this study. Data sharing is not applicable to this article.

## Supplementary Materials

The following supporting information can be downloaded at https://www.mdpi.com/article/10.3390/healthcare13101092/s1, Table S1: Trauma reintegration process for dissociation—multiply traumatized client; Table S2: trauma reintegration process for dissociation—rape; Table S3: Trauma reintegration process for dissociation—war trauma.
