# Supplementary material for: Using the Trauma Reintegration Process to Treat Posttraumatic Stress Disorder with Dissociation and Somatic Features: A Case Series
Source: Healthcare (Basel). 2025 May 8;13(10):1092. doi: 10.3390/healthcare13101092 (PMC12111324; doi:10.3390/healthcare13101092)
Supplement: Supplementary file 1 [file healthcare-13-01092-s001.zip › healthcare-3567113-supplementary.pdf]

Appendices: Supplemental Tables

Supplemental Table S1: Trauma Reintegration Process for Dissociation—Multiply Traumatized Client
Supplemental Table S2: Trauma Reintegration Process for Dissociation—Rape
Supplemental Table S3: Trauma Reintegration Process for Dissociation—War Trauma

Supplemental Table S1: Trauma Reintegration Process for Dissociation—Multiply Traumatized Client

	**Steps for EFT/tapping practitioner**	**Examples of client responses**
Step 1: Remember	Once the client is stable and comes in with a present-day issue where the SUD level will not lower, ask questions to link the distress of today to a earlier time when they had this same feeling in the body. Trace this back in time and probe the client's point of view.	Therapist: Where do you see yourself? What are you noticing? Client: I am in my old house, I am looking out the window. My mom just left me alone with my brothers. I see her leaving.
Step 2: Reunite	Guide the client to image getting as close as possible to the part of themselves that they see still stuck in the trauma. If SUD score increases, use EFT. Ask the client what they feel, hear, smell. Guide client through as many rounds of EFT as needed to lower the SUD score, moving closer and closer to the younger part, explaining and comforting the dissociated self with the truth of how the trauma is now over.	Therapist: Bring the you of today, the adult you who knows you are safe now, back into that house. Go up to the one in the house.<br>Client: She is terrified, she won't let me near her.<br>Therapist: Use EFT.<br>Client: She is calmer, looking at me. Therapist: Go closer.<br>Client: I can reach her now. Therapist: See if you can pick her up, comfort her.<br>Client: I have her. I tell her Mom came back, she did a good job, she is safe. She is shaking.<br>Therapist: Use EFT. Client: Now she is crying. Therapist: Use EFT.<br>Client: She is calmer. She is in my arms.
Step 3: Reintegrate	After the SUD level has lowered, invite the dissociated part (the younger self) forward to today to look out the client's eyes in the present day. Repeat EFT.	Therapist: Would she like to see how your life turned out? Client: Oh yes.<br>Therapist: Close your eyes and bring her forward in your life, show her how you finished school, have a son, have your own home. Show her the grandchildren. When you get all the way to today, have her look out your eyes and see today. Look around the room.<br>Client: I am here, happy, safe. Therapist adds another round of tapping till the SUD score is 0 and client is in present time.

Supplemental Table S2: Trauma Reintegration Process for Dissociation—Rape

	**Steps for EFT/tapping practitioner**	**Examples of client responses**
Step 1: Remember	Ask questions to probe the client's point of view.	Therapist: Where do you see yourself, what are you noticing? Client: I am above the bed, in the corner of the room. I am drugged and don't know what is happening.
Step 2: Reunite	Guide the client to imagine getting as close as possible to the part of themselves that they see still stuck in the trauma. If SUDS score increases, use EFT. Ask the client what they feel, hear, smell. Guide client through as many rounds of EFT as needed, moving closer and closer to the younger part, explaining and comforting the dissociated self with the truth of how the trauma is now over.	Therapist: Bring the you of today into that room and to the you in the corner and tell her what happened.<br>Client: I can see it. I'm afraid. I don't want this to be.<br>Therapist: Yes, I hear you. Tell her the truth.<br>Client: It did happen. I thought he was my friend.<br>Therapist: Put your arms around her. Comfort her. Use EFT.<br>Client (crying): It is real.<br>Therapist: Let's go to the part of you in the bed now.<br>Client: I feel numb.<br>Therapist: Use EFT.<br>Client: Now I feel feel what is happening. Therapist: Use EFT. Now what do you feel?<br>Client: I want to kill him. Therapist: Feel the rage. What do you want to do?<br>Client: Push him off, scream.<br>Therapist helps client to give voice, stand up, or take any action client wishes. Then use EFT.<br>Client: I feel better. Calmer. Therapist: Let's get her out of there.
Step 3: Reintegrate	After the SUD level has lowered, invite the dissociated part (the younger self) forward to today to look out the client's eyes in the present day. Repeat EFT.	Therapist: With eyes closed, walk the part of you out of that room, out the door of that house, and show her how life turned out, how you are married today, have three children. When she is ready, invite her to look out your eyes and see today.<br>Client: It is over. I'm safe now. Therapist adds another round of tapping until the SUD level is a 0 and client is present in today.

Supplemental Table S3: Trauma Reintegration Process for Dissociation—War Trauma

	Steps for EFT/tapping practitioner	Examples of client responses
Step 1: Remember	Ask questions to probe the client's point of view.	Therapist: Where do you see yourself, what are you noticing? Client: I am in Afghanistan. There is a horrible smell. We come upon the Afghans who helped us. They have been decapitated.<br>Therapist: Use EFT.
Step 2: Reunite	Guide the client to imagine getting as close as possible to the part of themselves that they see still stuck in the trauma. If SUD score increases, use EFT. Ask the client what they feel, hear, smell. Guide client through as many rounds of EFT as needed, moving closer and closer to the younger part, explaining and comforting the dissociated self with the truth of how the trauma is now over.	Therapist: Bring the you of today who knows this time is over to the younger you.<br>Client: He is in shock. He is frozen, can't move. He can't cry.<br>Therapist continues rounds of EFT until all of these emotions have been felt in the body and honored.<br>Therapist: What happens next? Client: We bury the bodies.<br>Client begins to cry. Therapist allows him to speak, share, cry. Then use EFT. SUD score lowers but not to 0 as rage surfaces.<br>Client: I want to kill everyone now. Therapist validates the client, then uses EFT to lower the SUD score.<br>Therapist: Do it for your family, yourself, so you don't carry this hate and rage.<br>Client: I feel calmer now. I still see myself standing there.<br>Therapist: Go closer to the younger you. What can you tell that younger you?<br>Client: I'm sorry I had to go through that. I did the best I could. I can leave this time. Client is now crying. Therapist allows this emotion and then uses another round of tapping to release grief.<br>Therapist: Let's get him out of there.
Step 3: Reintegrate	After the SUD level has lowered, invite the dissociated part (the younger self) forward to today to look out the client's eyes in the present day. Repeat EFT.	Therapist: With eyes closed, bring the you from Afghanistan to today. When he is ready, invite him to look out your eyes and see today.<br>Client: The war is over. I can honor in another way the men who were killed. Do EFT until SUD score is 0 and client is in present time.
